# Congenital Gastric Outlet Obstruction and Nonimmune Hydrops Fetalis: A Prenatal Sonographic Diagnosis of a Case with Hydrothorax and Ascites

**DOI:** 10.1100/tsw.2008.114

**Published:** 2008-09-30

**Authors:** A. Yisau Abdulkadir, O. A. M. Adesiyun, A. Adisa Fawole, A. Peter Aboyeji

**Affiliations:** ^1^ Department of Radiology, University of Ilorin Teaching Hospital, Ilorin, Nigeria; ^2^ Department of Obstetrics and Gynaecology, University of Ilorin Teaching Hospital, Ilorin, Nigeria

**Keywords:** anasarca, cardiac arrythmia, fetal ascite, gastric outlet obstruction, hydrops fetalis, prenatal ultrasonography

## Abstract

A case of a male fetus with sonographic diagnosis of hydrops fetalis at 19-week gestation is reported. The fetus had anasarca, bilateral massive pleural effusion, and ascites, in addition to cardiac arrhythmia and congenital gastric outlet obstruction. Mother's clinical history and laboratory workup excluded immune hydrops. The etiological dilemma and fetal outcome are discussed. We concluded, based on this case, that when fetal hydrops occurs early and is associated with multiple congenital anomalies, prolonging the pregnancy may be futile.

## INTRODUCTION

Hydrops fetalis, immune or nonimmune, continues to be a management challenge. The mortality is high even at the best centers despite advances in medicine[1,2,3,4]. Over the years, the contribution of rhesus isoimmunization to hydrops fetalis has declined considerably and nonimmune hydrops now accounts for about 80% of cases, with a gamut of causes that includes chromosomal abnormalities, cardiac anomalies, tumors, twin-twin transfusion syndrome, and metabolic abnormalities[1,2,3,4,5]. However, in 15–50% of cases, no cause can be found irrespective of the extensiveness of investigations[1,2,3,4,5].

Prenatal diagnosis has been made possible by ultrasonography and magnetic resonance imaging. These modalities are relatively safe prenatally because of their nonionization. Serial imaging may be required to establish the diagnosis and the extent of the hydrops. Following which, extensive clinical workup in an attempt to identify the specific etiology is required. The search for etiology in suspected nonimmune hydrops fetalis (NIHF) starts with maternal clinical history directed to hereditary or metabolic diseases, diabetes, infections, anemias, and the drugs used[6]. Laboratory investigations, such as indirect Coombs test, to exclude immune causes; full blood counts and indices to exclude thalassemias; maternal blood chemistry testing for G-6-PD deficiency; Betke-Kleihauer testing for fetal-maternal transfusion; and screening for TORCHS (toxoplasmosis, rubella, CMV, herpes simplex, and syphilis)

infection during intrauterine pregnancy have invaluable uses in the search for etiology. Occasionally, invasive fetal testing is performed by means of amniocentesis or cordocentesis. Both methods pose a risk of fetal death[6].

A case of NIHF diagnosed by prenatal ultrasound having massive ascites and pleural effusion, in association with gastric outlet obstruction and cardiac arrhythmia, is reported.

## CASE HISTORY

A 19-year-old woman, gravida 2 para 1, who was certain of her last menstrual period, presented at the booking clinic with a 19-week uneventful pregnancy. Physical examination revealed no abnormality. However, there was wide disparity between the menstrual estimated gestational age and symphysio-fundal height. These were 19 weeks and 29 weeks, respectively. Her urinalysis and full blood count results were normal. She is blood group O Rh positive and genotype is AA. Screening for TORCHS infection using direct enzyme-linked immunosorbent assay (ELISA) was negative. Immediate ultrasound scan showed a single live fetus with cardiac arrhythmias (uncoordinated fetal cardiac activities with missed beats), fetal ascites, and polyhydramnios. The biparietal diameter (BPD) and fetal femur length (FL) measurements were both equivalent to gestational age of 19 weeks, 2 days.

She was scheduled for serial ultrasound monitoring, which was hampered by her need to travel and discuss with her employer. She returned 8 weeks later with complaints of absent fetal movement and lower abdominal pain not associated with fever, dysuria, or change in bowel habit. Blood pressure (100/60 mmHg) and urinalysis were normal. Abdominal ultrasound this time showed a lifeless fetus with gross anasarca, ascites, and hydrothorax. The fetal stomach was fluid distended and ended blindly, in keeping with gastric outlet obstruction (Fig. 1A). The lungs were solid and hypoplastic. The fetal kidney and the urinary bladder were normal. Associated findings include polyhydramnios (amniotic fluid index of 248/4), edematous fetal scalp, thickened edematous diaphragm, and minimal ventricular dilatation, but no spinal anomaly (Fig. 1B). There was no vertebral anomaly. Both BPD and FL correlated to 27 weeks, 1 day gestation. The cervix was dilated. An assessment of hydrops fetalis with intrauterine fetal death in active preterm labor was made. She spontaneously expelled a fresh stillbirth male fetus weighing 2.1 kg. The fetus had no dysmorphic feature aside from generalized anasarca. There were no malformations of the anus, placenta, or umbilical cord, and mother's indirect Coombs test was negative. Pathological confirmation was not feasible because postmortem examination of the fetus was refused as taboo by the patient's relatives.

## DISCUSSION

Hydrops fetalis consists of an abnormal accumulation of fluid in two or more fetal compartments, including ascites, pleural effusion, pericardial effusion, and skin edema[6,7]. Either fetal ascites or fetal pleural effusion is an uncommon anomaly[3,8]. Both coexisted with anasarca in this fetus (Figs.1A and B). Myriads of etiologies have been reported for NIHF, among which are cardiovascular, renal, and gastrointestinal anomalies. Others are infection, anemia, drugs, and inborn errors of metabolism. There has also been a reported case of an association with congenital hypothyroidism[7].

The precise etiology in this fetus was unknown. However, the possibility of an immune cause was unlikely because the mother was healthy, blood group O positive, and her indirect Coombs test was negative. Again, since she had normal full blood count and tested negative for ELISA tests, congenital infections (TORCHS) were an unlikely etiology in this case. Although not available at our center, where available, polymerase chain reaction (PCR) is a better option[6]. Cardiac arrhythmias and gastric outlet obstruction were the only identified fetal anomalies that could have contributed to the NIHF in this case. The polyhydramnios may be partly due to gastrointestinal malformation (gastric outlet obstruction and intestinal atresia). However, we found no case of gastric outlet atresia *in utero* reported in association

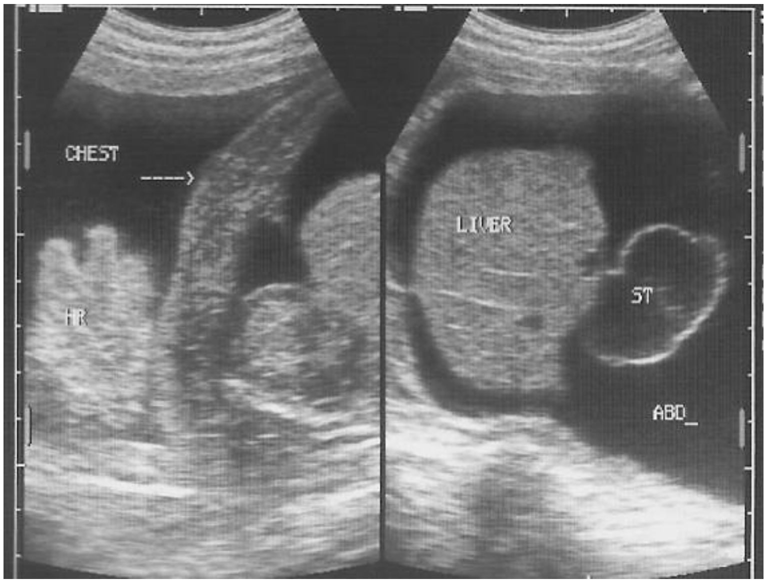

**A**

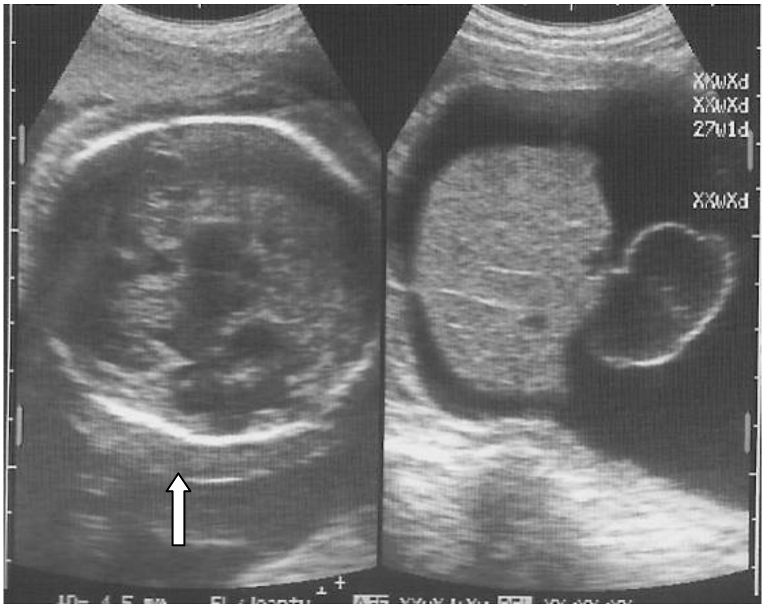

**B**

**FIGURE 1.** Sonogram of a 27-week-old male fetus with NIHF. (A) Longitudinal scans of fetal chest (left) and abdomen (right) showing hydrothorax, edematous diaphragm (arrowed), ascites, and the liver, with fluid-filled blind-ended stomach (ST). (B) Axial scan of the skull and longitudinal abdominal scan of the same fetus. Note the scalp edema (arrowed) and ventriculomegaly.

with fetal pleural effusion. Whether this is part of VACTERL (VATER syndrome or VACTERL association is a nonrandom association of birth defects), malformation is uncertain. However, clinical examination of the fetus shows normal anal opening and no limb anomaly. Although there was no plain radiographic workup, ultrasonography shows no vertebral anomaly.

Ultrasonography, which assisted in the diagnosis in this fetus, has a major role in determining the optimal approach to perinatal management of hydrops fetalis, and has contributed to a reduction in the perinatal mortality and morbidity associated with this disorder[8,9,10,11]. Similarly, magnetic resonance imaging, although not used in this case due to nonavailability, could assist in better delineation of the anatomy of such fetuses.

Fetal hydrothorax may present a wide spectrum of severity ranging from small, harmless effusions, to life-threatening thoracic compression[5,6,7]. The overall mortality is high and is even higher when associated with hydrops, gestational age less than 35 weeks at delivery, and bilateral effusions. Similarly, the findings in this case of fetal ascites at 19-weeks gestation, intrauterine fetal demise at 27 weeks, and the subsequent preterm labor are consistent with several authors' reports that the prognosis for fetal and neonatal survival is poor, especially where ascites developed before 24-weeks gestation[5,6,7,8].

There have been few reported cases of spontaneous resolution of fetal pleural effusion and fetal ascites[3,5,6,7,8,9]. When it does occur, it confers a good prognosis and better perinatal outcome for those fetuses without hydrops than those presenting with hydrops[3,8]. Intrauterine interventions, such as repeated thoracocentesis, thoracoamniotic shunting, and pleurodesis, have been shown to slightly improved chances of survival[3,4,10].

According to Abrams et al.[1], pregnancies with NIHF might result in abortion, intrauterine fetal death, or therapeutic termination, and delivering such fetuses early to treat worsening hydrops may not improve survival rates. Thus, the option of early delivery was not considered in this case due to cardiac arrhythmias and gastric atresia in association with gross fetal ascites, hydrothorax, and anasarca as early as 19 weeks of gestation. Aside from the fact that we lack facilities to cope with the challenges of ensuring fetal survival at such gestational ages, the cardiac and gastrointestinal anomalies were enormous challenges superimposing on prematurity.

The earlier the diagnosis of hydrops fetalis, the poorer the prognosis. The worsening of the hydropic features, ascites, pleural effusion, and polyhydramnios in this case may explain the preterm delivery and the fetal demise, probably from cardiac tamponade. Thus, prolonging pregnancy of a hydropic fetus with coexisting hydrothorax and ascites in the presence of cardiac arrhythmia and gastrointestinal anomaly may be futile.
